# Reduced expression of multiple gap junction proteins is a feature of cervical dysplasia

**DOI:** 10.1186/1476-4598-4-31

**Published:** 2005-08-09

**Authors:** Trond Aasen, Sheila V Graham, Mike Edward, Malcolm B Hodgins

**Affiliations:** 1 Squamous Cell Biology and Dermatology, Division of Cancer Sciences and Molecular Pathology, Robertson Building, University of Glasgow, 56 Dumbarton Road, Glasgow, G11 6NU, Scotland, UK; 2 Institute of Biomedical and Life Sciences, Division of Virology, University of Glasgow, Church Street, Glasgow, G11 6JR, Scotland, UK; 3 Centre for Cutaneous Research, Institute of Cell and Molecular Science, Queen Mary University of London, 4 Newark Street, Whitechapel, London E1 2AT, UK

**Keywords:** cervical cancer, gap junctions, connexins, papillomavirus, keratinocytes

## Abstract

Cervical dysplasia is a premalignant lesion associated with human papillomavirus (HPV) infection which, over time, can turn cancerous. Previous studies have indicated that loss of gap junctions may be a feature of cervical cancer and premalignant dysplasia. Loss of the gap junction protein connexin43 has been demonstrated in dysplastic cervix, but other connexins have not been investigated. In contrast we previously showed that HPV-associated cutaneous warts – and other hyperproliferative skin conditions – display a dramatic upregulation of certain connexins, in particular connexin26. By performing immunofluorescence staining after antigen retrieval of paraffin-embedded cervical tissue samples, this study reports for the first time that connexin26 and connexin30, in addition to connexin43, are expressed in differentiating cells of normal human cervical epithelia. Moreover, in dysplastic ectocervix, all connexins studied display a dramatic loss of expression compared to adjacent normal epithelia. The role of connexins in keratinocyte differentiation and carcinogenesis is discussed.

## Findings

Connexins, a family of 20 trans-membrane proteins in humans, comprise the main subunits of gap junctions – specialised clusters of intercellular channels that allow adjacent cells to directly share ions and hydrophilic molecules of up to ~1 KDa in size [1]. Gap junctional intercellular communication (GJIC) is thought to control tissue homeostasis and to coordinate cellular processes such as proliferation, migration and differentiation. Disruption of GJIC or mutations in connexins is associated with several human diseases such as hearing loss, neuropathies and various skin conditions [2].

There is also substantial evidence that connexins have a tumour suppressor role [reviewed in [3]]. While reduced or aberrant GJIC or connexin expression has been found in some tumours and in many tumour cell lines [4-7], restoration of GJIC in tumour cell lines by connexin transfection can reduce growth and tumourigenicity [8-10]. However, the tumour suppressive effects may be tissue and connexin-specific [11,12] and also appear to involve non-gap junctional properties of connexins [13-15]. Moreover, it has been observed that connexin expression (especially connexin26) is often upregulated in hyperplastic tissues including psoriatic epidermis and viral warts [16], benign prostatic hyperplasia [17], and mouse papillomas [18]. While induction of connexin26 and connexin43 has also been observed in metastatic breast carcinomas [19], others have reported that connexin26 and connexin43 are downregulated in mammary carcinoma cell lines and re-expression of these connexins leads to repression of tumour-forming ability [20]. Although potent tumour promoters markedly downregulate GJIC in cultured cells [21], intact skin painted with tumour promoters such as 12-O-tetradecanoylphorbol 13-acetate (TPA) show a dramatic upregulation of connexin26 and connexin43 expression [22-24]. Moreover, several reports have shown a negative correlation between expression of connexins and cell diapedesis and or tumour metastasis, including brain tumours [25,26], melanoma [27], breast carcinoma [28] and lung squamous cell carcinomas [29]. Thus, although connexins act as tumour suppressor genes in several types of cancers, the role of connexins in metastasis are more conflicting.

The association of certain "high risk" human papillomaviruses (HPVs) with the development of cervical cancer on the other hand has been clearly demonstrated [30], with several targets and functions of the viral oncoproteins identified [31]. However, tumour progression only occurs in a very small subpopulation of HPV infected individuals; thus, it is thought that several molecular and cellular changes are required over time for malignant conversion to take place. One of these cellular changes may be loss of connexin expression and/or GJIC. Indeed, it was observed more than four decades ago, using freeze-fracture electron microscopy, that normal cervix has abundant gap junctions, and that these are deficient in cervical carcinomas [32]. Further work also demonstrated a dramatic decrease of gap junction plaques in pre-malignant conditions such as severe dysplasia [33]. More recently, immunohistochemistry of cervical biopsies showed reduced connexin43 expression in dysplastic regions compared to normal epithelia [34] and work *in-vitro *has suggested that loss of GJIC may be an early event in papillomavirus associated cell transformation [35-38]. However, this may be limited to certain cell types or to particular oncogenes that are not always expressed during tumour progression (for example the E5 oncogene product that is frequently deleted after viral integration). Recently, we also showed that human cervical keratinocytes harbouring HPV-16 expressed several connexins (including connexin26, connexin30 and connexin43), and displayed extensive GJIC that was only lost at a late malignant stage [39]. This, together with our observation of a dramatic upregulation of connexin26 expression in cutaneous HPV warts [16], prompted us to investigate the expression pattern of several connexins in normal and dysplastic cervical epithelia.

Six duplicate slides of HPV-16 positive dysplastic cervix (cervical intraepithelial neoplasia (CIN) I, CIN I/II, and CIN III) were obtained from Dr. John Doorbar (Dr Karl Sotlar Institute for Pathology, Tubingen Germany, as previously described [40]). Antigen retrieval using pressure cooking in citrate buffer was performed followed by immunofluorescence staining using antibodies and methods as previously described [39]. All antibodies were diluted in PBS containing 1% bovine serum albumin (BSA), 0.1% Tween-20 and 0.01% SDS, and incubated overnight at 4°C. The presence of BSA and SDS was used to improve the connexin staining as previously shown for other antibodies [41]. Sections were mounted with Vectashield mounting medium containing DAPI and all images were obtained using an upright Zeiss Axioplan 2 fluorescence microscope. After immunofluorescence imaging the slides were washed and stained with Haematoxylin and Eosin (H&E) to verify previous CIN gradings.

**Figure 1:**
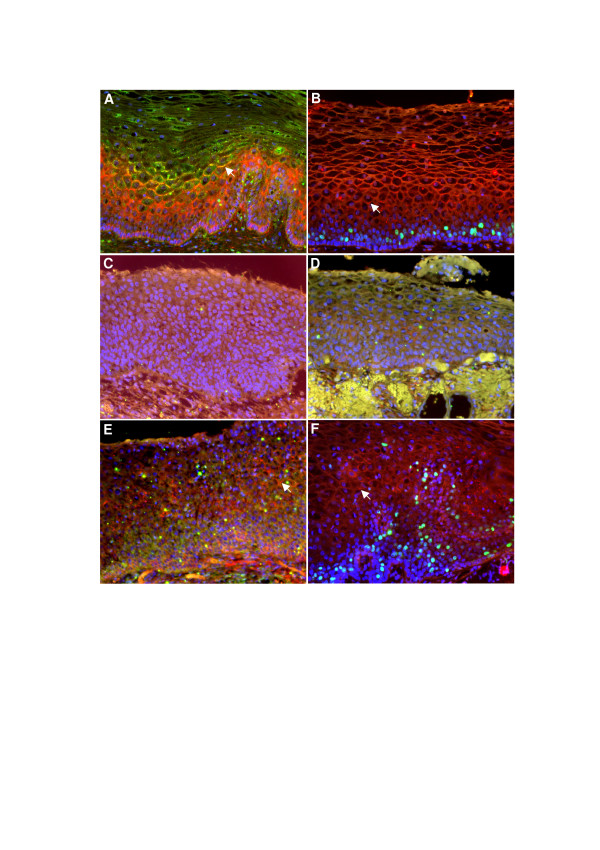
**Connexin expression in normal and dysplastic human cervical epithelium**. All nuclei are stained with DAPI (blue). **A: **All normal ectocervical tissue sections revealed positive staining of connexin43 gap junctions (red) particularly in the spinous layers. Connexin26 was also positive (green), particularly in upper spinous and more superficial layers. Overlapping expression of connexin43 and connexin26 was observed (yellow) and these areas displayed particularly large gap junction plaques (arrow). **B: **Connexin30 (red) displayed a similar staining pattern to Connexin26 although gap junction plaques were more frequently observed in lower spinous layers (arrow). In these normal sections, the proliferation marker Ki-67 was detected (turquoise) in basal and immediate suprabasal layers only, as expected. **(C) **In all premalignant CIN III sections there was complete absence of connexin26 (green) and connexin30 (red). **D: **In one CIN III lesion, small amounts of connexin43 (red) were present, mainly as diffuse cytoplasmic staining, but no positive connexin26 staining was detectable (green). **E: **In CIN I/II lesions no clear connexin26 gap junctions were present (green) whereas several connexin43 plaques were visible (arrow), but in a less homogenous fashion compared to normal ectocervix. **F: **Connexin30 gap junctions were clearly detected in areas of low grade CIN I lesions, although they tended to occur in stratified regions of cell differentiation (arrow) rather than areas of abnormal cellular proliferation (Ki-67, turquoise) and atypical cellular crowding.

As seen in Figure 1A, all sections of normal ectocervix expressed connexin43 (red) mainly in the suprabasal spinous layers. Some staining was also seen in the basal layer although it was difficult to identify specific gap junction plaques. Connexin26 and connexin30 were found mainly in the upper spinous layers, but some was also detected in the lower layers similar to connexin43, particularly with connexin30 (1A and 1B). Overlapping co-localisation (yellow gap junction plaques) was seen in connexin26/connexin43 triple-immunofluorescence staining experiments (Figure 1A) as observed in palmoplantar skin [42]. The more differentiated cervical squames were negative for all three connexins. Moreover, no definite connexin membrane plaques could be identified in the basal layer although some gap junction plaques are likely to be present as previously shown in early freeze-fracture electron microscopy studies [32,33]. As expected, the proliferation marker Ki-67 was detected in basal or suprabasal keratinocytes but not in differentiated cells (Figure 1B).

In contrast, all sections of dysplastic cervix displayed loss of protein expression of all three connexins investigated (Figure 1C–F). Connexin26 and connexin30 expression was completely absent in the CIN3 lesions (Figure 1C). In some areas however, particularly using high magnification power and high laser exposure, some connexin43 plaques could be seen in dysplastic areas, although it was difficult to assess accurately the dysplasia in those exact areas (Figure 1D–E). Some small connexin30 gap junction plaques were also identified in dysplastic tissue, but generally these appeared separate from cells positive for the proliferation marker Ki-67 in stratified cells of dysplastic cervix (Figure 1F). Thus, although some connexin30 and connexin43 gap junction plaques were occasionally seen in low grade CIN I/II cervical biopsies, there was always a dramatic loss of connexin expression compared to adjacent normal tissue.

In conclusion, this report demonstrates for the first time the presence of connexin26 and connexin30 gap junction plaques, as well as connexin43, in human ectocervix, displaying a similar staining pattern to that observed for connexin26 in vaginal epithelium [16]. Moreover, dysplastic areas of the biopsies show a dramatic loss of gap junction plaques, primarily due to reduced expression rather than cytoplasmic accumulation of connexin proteins. However, it cannot be excluded that cytoplasmic connexins are undetectable by the current staining protocol and antibodies available. The use of paraffin embedded tissue may reduce the detection efficiency of smaller gap junction plaques and/or cytoplasmic proteins. Some general background immunofluorescence is present in both normal and dysplastic tissue and this also hampers detection of cytoplasmic proteins. Significantly however, lack of staining in dysplastic lesions cannot be attributed to sample or patient factors, as the staining of both normal and dysplastic tissue were performed using the same tissue biopsies. It thus also appears that the lack of gap junctions in CIN lesions are likely to be controlled in an intracrine fashion rather than as an effect of paracrine or hormonal fashion, particularly since areas of gap junction plaques where observed in differentiating cells layered in between dysplastic/Ki-67 positive cells. Currently very little is known regarding how connexin gene expression is regulated and further studies, perhaps using laser capture followed by RT-PCR and/or methylation specific PCR, are required to elucidate what regulates the overexpression and loss of connexins, in cutaneous warts and CIN lesions respectively. Our previous work on model cell lines indicate that a combination of cytoplasmic connexin accumulation and loss of connexin transcription may occur [39].

Expression of connexin26 but not connexin40 and connexin43 has been shown to reduce tumourigenesis of HeLa cells (cervical tumour cells) [12]. It is also likely that other connexins are expressed in human ectocervix, for example connexin31 which is expressed in differentiating keratinocytes in the epidermis [43], and may play a different role in epidermal biology and carcinogenesis. In order to answer some of these outstanding questions, it is imperative to decipher more accurately what biological role gap junctions and individual connexins execute.

These results are clearly in disparity to observations in interfollicular epidermis, where connexin26 and connexin30 are normally not present (apart from palmoplantar skin) but becomes highly expressed in areas of hyperproliferation such as viral warts [16]. The reason for this remains unknown, but several factors may contribute. For example, the stratifying cervical epithelium is of non-keratinising nature. Unlike dysplastic cervical epithelia, cutaneous warts are typically highly proliferative, thick lesions, and there is some evidence that the presence of gap junctions may favour such stratification (perhaps due to metabolic co-operation) [39]. Conversely, it is currently difficult to explain why gap junctions are lost in HPV-16 positive CIN lesions. It is clear however that basal keratinocytes express at least tenfold fewer gap junction plaques than differentiated keratinocytes [32,33], and the lack of gap junctions in CIN lesions may simply reflect failure of keratinocyte differentiation in the stratified layers. Although there is some evidence, it remains to be seen whether connexins themselves play a direct role in regulating keratinocyte differentiation. Loss of connexin expression may also be associated with HPV infection, although our recent results suggests that such correlation requires high levels of oncogene expression and/or a further malignant progressed state [39]. A viral advantage associated with loss of GJIC has not been described, however a recent intriguing report has documented gap-junction-mediated immunological coupling allowing direct transfer of antigenic peptides [44] which may be involved in ensuring proper antigenic T-cell response against viruses such as HPV hiding in cells or expressing anti-apoptotic proteins.

## Authors' contributions

TA carried out all experimental assays and drafted the manuscript. TA, MBH, ME and SVG participated in study design and coordination, data interpretation and manuscript preparation. All authors read and approved the final manuscript.
